# A Novel CGM Metric-Gradient and Combining Mean Sensor Glucose Enable to Improve the Prediction of Nocturnal Hypoglycemic Events in Patients with Diabetes

**DOI:** 10.1155/2020/8830774

**Published:** 2020-11-02

**Authors:** Jingzhen Li, Xiaojing Ma, Igbe Tobore, Yuhang Liu, Abhishek Kandwal, Lei Wang, Jingyi Lu, Wei Lu, Yuqian Bao, Jian Zhou, Zedong Nie

**Affiliations:** ^1^Shenzhen Institutes of Advanced Technology, Chinese Academy of Sciences, Shenzhen 518055, China; ^2^Department of Endocrinology and Metabolism, Shanghai Clinical Center for Diabetes, Shanghai Diabetes Institute, Shanghai Jiao Tong University Affiliated Sixth People's Hospital, Shanghai 200233, China

## Abstract

Nocturnal hypoglycemia is a serious complication of insulin-treated diabetes, and it is often asymptomatic. A novel CGM metric-gradient was proposed in this paper, and a method of combining mean sensor glucose (MSG) and gradient was presented for the prediction of nocturnal hypoglycemia. For this purpose, the data from continuous glucose monitoring (CGM) encompassing 1,921 patients with diabetes were analyzed, and a total of 302 nocturnal hypoglycemic events were recorded. The MSG and gradient values were calculated, respectively, and then combined as a new metric (*i.e.*, MSG+gradient). In addition, the prediction was conducted by four algorithms, namely, logistic regression, support vector machine, random forest, and long short-term memory. The results revealed that the gradient of CGM showed a downward trend before hypoglycemic events happened. Additionally, the results indicated that the specificity and sensitivity based on the proposed method were better than the conventional metrics of low blood glucose index (LBGI), coefficient of variation (CV), mean absolute glucose (MAG), lability index (LI), *etc.*, and the complex metrics of MSG+LBGI, MSG+CV, MSG+MAG, and MSG+LI, *etc*. Specifically, the specificity and sensitivity were greater than 96.07% and 96.03% at the prediction horizon of 15 minutes and greater than 87.79% and 90.07% at the prediction horizon of 30 minutes when the proposed method was adopted to predict nocturnal hypoglycemic events in the aforementioned four algorithms. Therefore, the proposed method of combining MSG and gradient may enable to improve the prediction of nocturnal hypoglycemic events. Future studies are warranted to confirm the validity of this metric.

## 1. Introduction

Diabetes mellitus is a metabolic disease resulting from defects in insulin secretion, insulin action, or both [1, 2]. Hypoglycemia is a common side effect of insulin therapy in diabetes, and it is one of the important limiting factors in the glycemic management of patients with diabetes [3]. It is estimated that 54.0% of patients with type 1 diabetes mellitus (T1DM) and 27.4% of patients with type 2 diabetes mellitus (T2DM) had nocturnal hypoglycemia and usually happened at around 1:00 am to 4:00 am [4, 5]. Furthermore, 75% of hypoglycemic events associated with coma or seizure occur at night because the autonomic symptoms are often not strong enough to awaken the patients [6]. Some studies reported that the “dead-in-bed” syndrome probably is the result of severe nocturnal hypoglycemia, which accounts for 5–6% of mortality cases in patients with T1DM [7, 8]. Therefore, the prediction of nocturnal hypoglycemia is urgent and important.

Continuous glucose monitoring (CGM) is regarded as the roadmap for 21st-century diabetes therapy [9]. One of the important potential benefits of CGM is that it can provide maximal information about glucose levels throughout the day. Thus far, a limited number of studies have been published utilizing the raw data from the CGM device to achieve hypoglycemia prediction. In a previous study by Mhaskar et al. [10], a certain percentage of patients were set as the training data, and the remainder of the patients was set as the test data. Furthermore, a deep neural network with ReLU nonlinearities was suggested to predict hypoglycemia. Similarly, a new scheme of a kernel-based regularization learning algorithm was proposed for hypoglycemia prediction in a previous study by Naumova et al. [11]. In addition, a real-time method combines five individual algorithms presented for hypoglycemia prediction in studies by Buckingham et al. [12, 13]. Meanwhile, considering that the glycemic variability is associated with hypoglycemia [14], some metrics which could reflect the glycemic variability and hypoglycemia were also presented to achieve the prediction of nocturnal hypoglycemia [15]. The conventional metrics include mean sensor glucose (MSG), standard deviations of sensor glucose (SD), coefficient of variation (CV), mean absolute glucose (MAG), low blood glucose index (LBGI), and lability index (LI). However, it is noteworthy that the variation amplitude is considered, whereas the variation trend is neglected in the aforementioned metrics. As well known, hypoglycemia is more likely to occur when blood glucose shows a decreasing trend rather than an increasing trend. In this paper, a novel CGM metric, which is named as gradient, was proposed in this paper. In terms of the definition of gradient, it can reflect the variation trend of blood glucose. In addition, the MSG can reflect the overall blood glucose level, which is associated with hypoglycemia. Therefore, a new method which combines MSG and gradient of CGM was proposed to predict nocturnal hypoglycemia in this paper. Four algorithms were adopted to evaluate the prediction performance at different prediction horizons.

## 2. Methods

### 2.1. Procedure

The study protocol was approved by the ethics committees of Shanghai Jiao Tong University Affiliated Sixth People's Hospital in accordance with the principles of the Declaration of Helsinki. Written informed consent was obtained from each participant before the measurement. The glucose value was obtained by using a commercial CGM system (iPro2, Medtronic Inc., Northridge, CA). The measuring range of the CGM system was from 2.2 mmol/L to 22.2 mmol/L. The monitoring interval of the CGM system was 5 minutes. Each patient had three-day CGM records, generating a daily record of 288 continuous glucose values. Initially, a total of 5,763 days of CGM records were collected from 1,921 patients. Furthermore, there were at least four capillary blood glucose readings per day measured by using a SureStep blood glucose meter (LifeScan, Milpitas, CA) to calibrate the CGM system. In this paper, we mainly focused on the investigation on nocturnal hypoglycemia. The reasons why the prediction of nocturnal hypoglycemia is considered in this work are as follows. Firstly, as mentioned above, nocturnal hypoglycemia has important clinical significance. Secondly, the nocturnal blood glucose level is less affected by diet, exercise, and other intervention factors, which helps to evaluate the prediction performance of the proposed metric. According to the bedtime and awakening time in most of the patients with diabetes, the nocturnal period for outcome assessment each night was from 22:00 to 06:00 of the following morning in this paper [16]. Therefore, generally, a record of 97 continuous glucose values was acquired each night for the patient. However, some CGM records might be incomplete or missing. The missing CGM value was interpolated by using a straight line estimation if only one data was missing. The CGM records were excluded from further analysis if two or more data in the record were missing. In this paper, 809 CGM records were excluded, and the remaining 4,954 CGM records were used for our study.

The nocturnal hypoglycemic event was defined as having at least 15 minutes of CGM values ≤ 3.9 mmol/L, and it occurred at night from 22:00 to 06:00 [17]. Hypoglycemic events were excluded from further analysis if they occurred <2 hours after a previous event. According to the aforementioned definition, 302 nocturnal hypoglycemic events from 4,954 CGM records were available for further study in this paper.

### 2.2. MSG and Gradient Calculation

In terms of the definition of gradient, it can represent the maximum along the direction derivative of a function at a point [18–20]. Assuming that the CGM data is expressed as a matrix of CGM = {*G*_1_, *G*_2_, *G*_3_, ⋯, *G*_*i*_, ⋯, *G*_*N*_}, the MSG and gradient of CGM data can be obtained by equation (1) and equation (2), respectively.

(1) $$\begin{array}{c} \text{MSG}=\frac{\sum_{i=1}^{N}G_{i}}{N}, \end{array}$$

(2) $$\begin{array}{c} \text{Gradient}\left(i\right)=\left\{\begin{array}{c} G_{i+1}-G_{i}\;i=1\\ \frac{G_{i+1}-G_{i-1}}{2}\;2\leq i\leq N-1\\ G_{i}-G_{i-1}\;i=N \end{array}\right., \end{array}$$

where *G*_*i*_ represents the *i*th value of CGM, Gradient(*i*) is the *i*th gradient value of CGM, and *N* is the number of CGM data.

The combination of MSG and gradient can be expressed as a matrix of {MSG, weight × gradient}. It is noted that the weight between MSG and gradient was adaptive when combining MSG and gradient. The weight of MSG was set as 1, and the weight of the gradient depended on the value of MSG. For instance, if the MSG value was equal to 4.9, the weight of the gradient would be set as 4.9.

### 2.3. Hypoglycemia Prediction Algorithm

Four classical algorithms, namely, logistic regression (LR), support vector machine (SVM), random forest (RF), and long short-term memory (LSTM), were adopted to achieve the prediction of nocturnal hypoglycemia in our work. Those algorithms were conducted by Python 3.5 based on the Keras platform. The principle and parameter setup of algorithms were briefly introduced as follows. LR is a predictive analysis that uses a logistic function to model variables [21, 22]. LR has been widely used in data mining, automatic disease diagnosis, economic prediction, and so on. The LR parameters were set as the default value. SVM is a supervised learning model used for classification and prediction. The decision boundary of SVM is the maximum margin hyperplane for learning samples [23]. The kernel of SVM was set as “linear,” and the penalty parameter C of the error term was 1.0 in this paper. RF is a classical classifier which consists of a number of decision trees [24, 25]. It uses bootstrap aggregating and a random subspace method to build multiple decision trees and merges them together to get a more accurate and stable prediction. The RF parameters of n_estimators, max_features, criterion, and n_jobs were set as 200, “auto,” “entropy,” and 200, respectively. LSTM is an artificial recurrent neural network architecture used in the field of deep learning [26, 27]. The characteristic of LSTM is to use a memory module instead of ordinary hidden nodes, which is helpful to ensure that the gradient does not disappear or explode after passing over many time steps. In this paper, the model consisted of 3 LSTM layers, and the dimension of output was 10, 8, and 2. The return sequence of layer 1 and layer 2 was set as “true.”

### 2.4. Evaluation Method

In this paper, the specificity and sensitivity were adopted to evaluate the prediction performance of nocturnal hypoglycemia by different metrics and algorithms at different prediction horizons. In particular, sensitivity and specificity are the statistical measures of the performance of a binary classification test that is widely used in medicine. The calculations are as equation (3) and equation (4).

(3) $$\begin{array}{c} \text{Sensitivity}=\frac{\text{TP}}{\text{TP}+\text{FN}}, \end{array}$$

(4) $$\begin{array}{c} \text{Specificity}=\frac{\text{TN}}{\text{TN}+\text{FP}}. \end{array}$$

As shown in equation (3), TP represents that the hypoglycemia was correctly identified as hypoglycemia, and FN represents that the hypoglycemia was incorrectly identified as nonhypoglycemia. Therefore, sensitivity can reflect the proportion of positives (*i.e.*, hypoglycemia) that are correctly identified. Similarly, TN is that the nonhypoglycemia was correctly identified as nonhypoglycemia, and FP is that the nonhypoglycemia was incorrectly identified as hypoglycemia. Hence, specificity can reflect the proportion of negatives (*i.e.*, nonhypoglycemia) that are correctly identified.

## 3. Results

### 3.1. Study Population

A total of 1,921 patients with diabetes (T1DM was 289, and T2DM was 1,632) were recruited from the Department of Endocrinology and Metabolism of the Shanghai Jiao Tong University Affiliated Sixth People's Hospital between January 2018 and March 2019. The male percentage of the enrolled participants was 57.6%. The mean ± standard deviation age of the enrolled participants was 59 ± 10 years, and they had a mean ± standard deviation diabetes duration of 11.5 ± 6.5 years. The percentages of patients using insulin, biguanide, sulfonylureas, DPP-4i, and *α*-glycosidase inhibitor during therapy were 71.1%, 39.2%, 21.5%, 8.06%, and 33.4%, respectively.

### 3.2. Gradient Analyses

**Figure 1:**
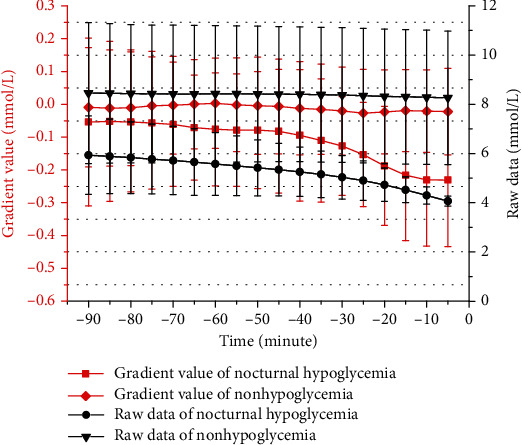
Raw data and gradient value from *t* = −90 minutes to *t* = −5 minutes before the nocturnal hypoglycemic events occurred at *t* = 0 minutes. The results in Figure 1 were presented as mean ± standard deviation.

Figure 1 demonstrates the raw glucose value and gradient value from *t* = −90 minute to *t* = −5 minute before the nocturnal hypoglycemic events occurred at *t* = 0 minute. The results in Figure 1 were presented as mean ± standard deviation. As shown in Figure 1, the raw glucose values showed a trend of approximately linearly declining before the hypoglycemic event occurred, whereas the raw glucose values remained almost unchanged with time for nonhypoglycemia. It could be observed that the gradient could reflect the variation direction of blood glucose. The gradient of hypoglycemic events showed a downward trend from -90 minutes to -5 minutes. For instance, the gradient value was -0.0611 mmol/L at -70 minutes, and it was less than -0.1099 mmol/L at -35 minutes. Furthermore, it was interesting to observe that the change of gradient value was more obvious in the time range -30 minutes to -5 minutes. The gradient value was about -0.2308 mmol/L at -5 minutes before the hypoglycemic events. However, the gradient value was almost the same from -90 minutes to -5 minutes for nonhypoglycemia, which was approximately -0.0015 mmol/L. In terms of the statistical analysis based on one-way ANOVA, the gradient value differed significantly across hypoglycemic events and nonhypoglycemia at different times (all *P* for trend <0.02).

### 3.3. Prediction Performance

Taking into account that the number of nocturnal hypoglycemic events was much less than the nonhypoglycemia and the importance of the sample balance in the train model, half of the nocturnal hypoglycemic events (*i.e.*, 151 events) and 151 data were randomly selected from the nonhypoglycemia and were used for training in four individual algorithms. The remaining data were used for verification.

Table 1 lists the specificity and sensitivity of different metrics and algorithms at different prediction horizons. The CGM length which was adopted for prediction was 30 minutes. The prediction horizons were 15 minutes, 30 minutes, 45 minutes, and 60 minutes. Both the single metric and complex metric were considered in our study. As illustrated in Table 1, it was interesting to observe that the prediction performance could be significantly improved when the proposed method of combining MSG and gradient (*i.e.*, MSG+gradient) was utilized. Specifically, the specificity was up to 97.12%, and the sensitivity was up to 98.01% at the prediction horizon of 15 minutes in the LR algorithm. Additionally, the specificity values were 90.83%, 82.42%, and 77.87%, and the sensitivity values were 90.07%, 90.07%, 90.73% at the prediction horizons of 30 minutes, 45 minutes, and 60 minutes, respectively. Furthermore, the results indicated that the prediction performance of the proposed method was not only better than the raw data and single metrics but also better than the complex metrics, including MSG+SD, MSG+CV, MSG+LAGE, MSG+MAG, MSG+LBGI, and MSG+LI at different prediction horizons. In addition, as shown in Table 1, the specificity and sensitivity of the proposed method were also the largest at the prediction horizons of 15 minutes and 30 minutes when other algorithms such as SVM, RF, and LSTM were used to achieve hypoglycemia prediction.

In order to analyze the influence of CGM length on the prediction performance, three different CGM lengths were considered in our work.  Table 2 lists the prediction results at the prediction horizon of 15 minutes and 30 minutes when the CGM lengths were 30 minutes, 45 minutes, and 60 minutes, respectively. It could be observed that the CGM length had little impact on the specificity and sensitivity when the method of combining MSG and gradient was used for prediction. For instance, the specificity values based on the LR algorithm were 90.83%, 91.18%, and 90.73% when the CGM length was 30 minutes, 45 minutes, and 60 minutes, respectively. Additionally, the prediction performance based on SVM, RF, and LSTM algorithm also revealed the similar conclusions.

To further understand the prediction performance of the proposed metric, the periods of declining sensor glucose without a hypoglycemic event were taken into account in our work. As shown in Figure 1, the average gradient from -90 minutes to -5 minutes was approximately -0.1119 mmol/L before the hypoglycemic events happened. Therefore, the data of nonhypoglycemia of which the average gradient was less than -0.1119 mmol/L was extracted for further analysis. A total of 1,438 data were obtained from nonhypoglycemia.  Table 3 demonstrates the false positivity rate (FPR) and false-negative rate (FNR) at the prediction horizon of 30 minutes MSG+gradient was adopted. The FNR values were 7.95%, 6.62%, 9.27%, and 7.95%, and the FPR values were 9.32%, 12.20%, 7.15%, and 7.69%, respectively, by four different algorithms. Therefore, it indicated that the proposed metric showed better FNR and FPR.

## 4. Discussion

This study revealed that the proposed method of combining MSG and gradient enabled to improve the prediction of nocturnal hypoglycemic events at different prediction horizons. The specificity was greater than 87.79%, and the sensitivity was greater than 90.07% at the prediction horizon of 30 minutes in four different algorithms in this paper. On the other hand, in the previous studies, the accuracy of hypoglycemic event prediction was 67.21% at the prediction horizon of 30 minutes and 81.75% at the prediction horizon of 20 minutes, respectively [10, 11]. In [12], using algorithms to shut off the insulin pump when hypoglycemia was predicted at the prediction horizon of 35 minutes, hypoglycemia was possibly prevented for 84%. In [28], a hypoglycemia prevention rate of nearly 80% after exercise was achieved by using the MiniMed 670G system. In [29], hypoglycemia was prevented in 76.8% of instances of predictive suspend. Compared with previous studies, the reason why the proposed method showed better prediction performance might be explained as follows. Firstly, the MSG value of CGM could reflect the overall blood glucose level over a period of time. As known to all, the lower the blood glucose value, the higher the probability of hypoglycemia. In addition, the occurrence of hypoglycemia was related to the change in blood glucose. A hypoglycemic event was more likely to occur when the blood glucose level showed a downward trend rather than an upward trend. The gradient value of CGM could reflect the direction of blood glucose variability. Therefore, the method of combining MSG and gradient was helpful to improve the prediction of hypoglycemia. It could be observed that the specificity and sensitivity based on the method of combining MSG and gradient were higher than the other metrics at different prediction horizons, especially in short prediction horizons of 15 minutes or 30 minutes. The reason might be associated with the change of gradient value. As shown in Figure 1, the closer the hypoglycemic event occurred, the greater the change of gradient value was. Thus, it indicated that the proposed method would show more obvious advantages in the short prediction horizon.

Our study is only a first step toward MSG and gradient and its application on nocturnal hypoglycemia. Three limitations of this study should be noted. Firstly, there were too few nocturnal hypoglycemic events to analyze, which requires a larger study of longer duration and participants. Secondly, the participants enrolled in this study were mainly patients with T2DM. However, compared with T2DM, patients with T1DM show a higher degree of glycemic variability, and the hypoglycemia predication may be more difficult. Since most of the enrolled subjects were T2DM, the results of our study might not be generalizable to all patients with diabetes, especially for T1DM. Therefore, a further study of the new metric by using a larger sample of patients with T1DM is necessary. Thirdly, the prediction of nocturnal hypoglycemia was static analysis in this work. The feasibility still needs to be evaluated when the proposed metric is used for real-time prediction of nocturnal hypoglycemia in patients with diabetes. A potential approach is that the metric can be integrated into wearable devices or CGM systems to verify the feasibility of the real-time hypoglycemia prediction. In the near future, we will try to incorporate the proposed metric and other factors including insulin dosage, carbohydrates intake, and daily activities to improve the specificity and sensitivity of hypoglycemia prediction. In addition, the feasibility of whether the proposed metric can be used in insulin pump to achieve the insulin suspension before hypoglycemia at different prediction horizons needs to be further investigated.

## Figures and Tables

**Table 1:** The specificity and sensitivity of the prediction of nocturnal hypoglycemic events based on different metrics and algorithms (CGM length = 30 minutes).

pH	Method	LR		SVM		RF		LSTM	
		SPE (%)	SEN (%)	SPE (%)	SEN (%)	SPE (%)	SEN (%)	SPE (%)	SEN (%)
15 min	Raw data	95.76	95.36	96.31	96.03	94.82	94.70	93.51	92.05
	MSG	85.89	90.07	86.06	90.07	80.21	90.07	86.06	90.07
	Gradient	52.00	90.73	54.51	89.40	49.02	90.07	51.32	89.40
	SD	24.24	91.39	26.83	89.40	13.53	89.40	22.17	91.39
	CV	69.75	76.82	89.42	54.97	40.84	90.07	39.94	92.72
	LAGE	21.37	91.39	21.37	91.39	16.15	90.73	21.37	91.39
	MAG	32.87	84.77	32.78	84.77	15.58	90.73	32.78	84.77
	LBGI	89.18	90.07	89.03	90.07	86.06	90.07	89.26	90.07
	LI	32.57	84.77	32.57	84.77	16.06	89.40	32.57	84.77
	MSG+gradient	97.12	98.01	97.40	98.01	96.07	96.03	96.88	97.35
	MSG+SD	93.61	94.70	93.82	94.04	92.42	92.72	94.13	94.04
	MSG+CV	87.81	90.07	88.43	90.07	93.02	93.38	87.61	90.73
	MSG+LAGE	94.26	94.70	94.67	94.70	93.00	94.04	94.18	95.36
	MSG+MAG	93.89	94.04	94.94	94.70	93.46	93.38	93.34	94.70
	MSG+LBGI	88.53	90.07	89.03	90.07	90.09	90.73	89.03	90.07
	MSG+LI	95.27	90.73	94.09	96.03	93.40	96.03	94.36	94.70

30 min	Raw data	83.70	90.07	85.43	90.07	83.99	90.07	85.50	90.07
	MSG	75.99	90.07	76.11	90.07	61.52	90.07	76.11	90.07
	Gradient	30.97	90.73	31.38	90.07	27.40	89.40	28.32	90.07
	SD	21.45	89.40	23.22	88.74	8.48	88.74	23.22	88.74
	CV	26.95	94.70	1.29	90.73	26.66	90.73	43.84	86.75
	LAGE	20.84	89.40	20.84	89.40	6.30	90.73	20.84	89.40
	MAG	31.75	82.12	31.76	82.12	17.17	90.07	43.91	69.54
	LBGI	82.92	90.07	83.12	90.07	81.38	89.40	83.34	90.07
	LI	42.70	70.20	16.84	92.05	10.08	88.74	31.49	82.12
	MSG+gradient	90.83	90.07	90.30	90.73	87.79	90.07	90.20	90.07
	MSG+SD	87.94	90.73	88.61	90.07	83.11	90.73	88.57	90.07
	MSG+CV	78.11	90.07	78.11	90.07	83.68	90.07	79.07	90.07
	MSG+LAGE	87.36	90.73	88.47	90.07	81.15	90.07	89.27	90.07
	MSG+MAG	86.02	90.07	88.15	90.07	79.79	90.07	87.53	90.07
	MSG+LBGI	79.26	90.73	77.00	90.07	81.24	90.07	81.34	90.07
	MSG+LI	87.99	90.73	88.48	90.07	78.93	90.73	79.58	90.07

45 min	Raw data	75.56	90.07	78.84	90.73	80.24	90.07	80.72	90.07
	MSG	65.66	90.07	65.46	90.07	54.54	90.07	65.11	90.07
	Gradient	12.05	90.73	11.09	91.39	21.34	90.07	11.43	90.73
	SD	20.58	89.40	22.42	88.08	7.03	90.07	19.91	89.40
	CV	75.75	57.62	10.82	60.93	26.56	90.73	53.56	78.81
	LAGE	19.95	89.40	19.94	89.40	7.77	89.40	19.95	89.40
	MAG	30.93	86.09	15.72	90.07	12.91	89.40	15.72	90.07
	LBGI	82.01	82.78	79.15	87.42	79.35	85.43	81.29	82.78
	LI	42.62	71.52	30.61	86.09	16.06	86.09	30.52	86.09
	MSG+gradient	82.42	90.07	81.15	90.73	79.79	90.07	82.84	90.07
	MSG+SD	81.79	91.39	82.14	90.07	74.84	90.07	82.19	90.07
	MSG+CV	65.84	90.07	66.98	90.07	73.32	90.07	67.02	90.07
	MSG+LAGE	81.55	90.73	81.95	90.73	72.27	91.39	81.32	90.73
	MSG+MAG	83.01	90.07	82.23	90.07	70.34	90.73	81.68	90.07
	MSG+LBGI	64.38	90.07	65.65	90.07	54.87	90.07	65.41	90.07
	MSG+LI	82.48	90.07	80.39	90.73	72.95	90.07	77.43	90.07

60 min	Raw data	71.68	90.07	70.65	90.73	64.49	90.73	70.19	90.07
	MSG	58.73	90.07	58.56	90.07	41.16	90.07	58.73	90.07
	Gradient	11.66	90.07	11.36	90.07	13.77	90.07	14.93	86.75
	SD	22.39	88.74	20.52	90.73	7.91	89.40	30.93	82.12
	CV	77.35	59.60	8.05	68.21	30.43	90.07	40.47	87.42
	LAGE	19.73	90.73	19.73	90.73	23.87	86.09	19.65	90.73
	MAG	30.62	84.11	30.63	84.11	21.83	87.42	30.64	84.11
	LBGI	81.43	80.13	82.32	78.15	79.45	79.47	80.71	80.79
	LI	49.75	67.55	30.33	84.11	18.29	86.09	41.69	73.51
	MSG+gradient	77.87	90.73	77.25	90.07	76.79	90.07	81.10	90.07
	MSG+SD	74.95	91.39	74.20	90.07	67.32	90.73	74.55	90.07
	MSG+CV	59.28	90.73	61.46	90.07	63.15	90.07	59.25	90.07
	MSG+LAGE	76.75	90.73	75.97	90.07	62.30	90.07	76.41	90.07
	MSG+MAG	77.75	90.73	78.85	90.07	69.74	90.07	76.89	90.73
	MSG+LBGI	57.46	90.07	58.71	90.07	45.14	90.07	58.10	90.07
	MSG+LI	76.51	90.73	76.67	90.73	70.87	90.07	78.90	90.07

PH: prediction horizon; SPE: specificity; SEN: sensitivity; LR: logistic regression; SVM: support vector machine; RF: random forest; LSTM: long short-term memory; MSG: mean sensor glucose; SD: standard deviations of sensor glucose; CV: coefficient of variation; LAGE: largest amplitude of glycemic excursion; MAG: mean absolute glucose; LBGI: low blood glucose index; LI: lability index. The CGM length which was used for the prediction of nocturnal hypoglycemic events was 30 minutes in this table.

**Table 2:** The influence of CGM length on the specificity and sensitivity of different algorithms when the metric of MSG+gradient was adopted.

pH	Algorithm	CGM length = 30 min		CGM length = 45 min		CGM length = 60 min	
		SPE (%)	SEN (%)	SPE (%)	SEN (%)	SPE (%)	SEN (%)
15 min	LR	97.12	98.01	97.47	97.70	96.85	97.35
	SVM	97.40	98.01	97.41	97.35	96.26	97.35
	RF	96.07	96.03	95.92	95.36	95.30	95.36
	LSTM	96.88	97.35	97.10	97.35	96.46	96.69

30 min	LR	90.83	90.07	91.18	90.07	90.73	91.39
	SVM	90.30	90.73	90.08	90.07	90.09	90.73
	RF	87.79	90.07	87.53	90.73	86.95	90.07
	LSTM	90.20	90.07	90.87	90.73	89.30	90.73

PH: prediction horizon; SPE: specificity; SEN: sensitivity; LR: logistic regression; SVM: support vector machine; RF: random forest; LSTM: long short-term memory.

**Table 3:** The FPR and FNR in the periods of declining sensor glucose without a hypoglycemic event when the metric of MSG+gradient was adopted at the prediction horizon of 30 minutes.

Algorithm	LR	SVM	RF	LSTM
FPR (%)	9.32	12.20	7.15	7.69
FNR (%)	7.95	6.62	9.27	7.95

FPR: false positivity rate; FNR: false-negative rate; LR: logistic regression; SVM: support vector machine; RF: random forest; LSTM: long short-term memory.

## Data Availability

Requests for access to these data should be made to zd.nie@siat.ac.cn or zhoujian@sjtu.edu.cn.
